# Ultrasonic-With-Ballistic Versus Pneumatic Lithotripsy in Percutaneous Nephrolithotomy for 2-4 cm Renal Calculi: A Prospective Quasi-experimental Comparative Study in a Bangladeshi Tertiary-Care Hospital

**DOI:** 10.7759/cureus.116404

**Published:** 2026-09-17

**Authors:** Md. Mahmudul Hasan, Md. Mezbahul Moker Rabin, Mohammad Alomgir, Md. Mahmood Hasan, Md. Al Mamun, Mahmud Ul Alam Mondal, Md. Rashedul Islam, Md. Shahinoor, Ujjal Barua

**Affiliations:** 1 Department of Urology, Rangpur Medical College Hospital, Rangpur, BGD; 2 Department of Urology, Chittagong Medical College Hospital, Chattogram, BGD

**Keywords:** ballistic lithotripsy, bangladesh, intracorporeal lithotripsy, percutaneous nephrolithotomy, pneumatic lithotripsy, renal calculi, stone clearance, ultrasonic lithotripsy

## Abstract

Background

Percutaneous nephrolithotomy (PCNL) is the standard treatment for large renal calculi, and the use of an intracorporeal lithotripter strongly influences its success. This study aimed to compare the effectiveness and safety of ultrasonic-with-ballistic lithotripsy (hereafter the combined device) and pneumatic lithotripsy during PCNL in a tertiary-care hospital in Bangladesh.

Methods

In this prospective, non-randomized quasi-experimental study, 52 adults aged 18-70 years with renal calculi 2-4 cm in size who underwent PCNL between January 2022 and December 2022 were enrolled and allocated to ultrasonic-with-ballistic lithotripsy (Group 1, n=26) or pneumatic lithotripsy (Group 2, n=26). The first patient was allocated by lottery and every subsequent patient alternately, so allocation was quasi-random and not concealed. The prespecified primary outcome was the stone-free rate on plain kidney-ureter-bladder radiography at four weeks, defined as no residual fragment or a single non-obstructing fragment smaller than 4 mm. Secondary outcomes were stone disintegration (fragmentation-and-extraction) time, total operative time, perioperative Hb drop, blood transfusion, complications and length of hospital stay. Postoperative complications were graded via the modified Clavien-Dindo classification. Categorical and continuous variables were compared via the chi-square test, Fisher’s exact test and Student’s t test; effect estimates are presented with 95% confidence intervals, and p<0.05 was considered statistically significant.

Results

The demographic and stone characteristics of the two groups were similar, although mean stone size was 2.4 mm smaller in Group 1 (p=0.068). The stone-free rate was significantly greater in Group 1 (25/26 (96.2%) versus 19/26 (73.1%); risk difference 23.1%, 95% CI 3.0% to 42.5%; relative risk 1.32, 95% CI 1.03 to 1.68; Fisher exact p=0.049). Stone disintegration time (22.1±5.9 versus 28.9±8.6 minutes; mean difference −6.8 minutes, 95% CI −10.9 to −2.7; p=0.002) and total operative time (57.7±15.6 versus 86.2±13.9 minutes; mean difference −28.5 minutes, 95% CI −36.7 to −20.3; p<0.001) were significantly shorter in Group 1. In contrast, the mean Hb drop (1.4±0.4 versus 1.6±0.5 g/dL; p=0.118), blood transfusion requirement (9/26 (34.6%) versus 12/26 (46.2%); p=0.397), postoperative fever (4/26 (15.4%) versus 3/26 (11.5%); p=0.668), overall complications, and length of hospital stay did not differ significantly between the groups.

Conclusions

Ultrasonic-with-ballistic lithotripsy was associated with a higher radiographically defined stone-free rate and with shorter disintegration and total operative times than pneumatic lithotripsy, and no statistically significant difference in the observed complications or in hospital stay was detected. Because allocation was not randomized, the sample was small, stone-free status was assessed radiographically rather than tomographically, and no cost analysis was undertaken, these findings describe an association rather than a causal effect and do not establish equivalence in safety. Adequately powered multicenter randomized trials with computed tomography-based stone-free assessment and formal cost-effectiveness evaluation are needed before routine adoption in resource-limited settings can be recommended.

## Introduction

Urolithiasis is among the most common urological disorders worldwide, and its prevalence has risen steadily over recent decades, affecting close to 1 in 10 people in many regions [1]. The burden increasingly falls on younger, working-age adults in developing countries, where large and staghorn calculi frequently present late and demand definitive surgical clearance. Since percutaneous nephrolithotomy (PCNL) was first described in 1976, it has become the standard of care for large and complex renal calculi, offering high stone-free rates with acceptable morbidity [2,3]. Progressive advances in instrument miniaturization, imaging and access techniques have broadened its indications and improved its safety so that PCNL is recommended as a first-line treatment for renal stones larger than 2 cm in appropriately selected patients [4-7].

Operative success in PCNL is multifactorial. It depends on the stone burden, density and configuration, on pelvicalyceal anatomy and the site and number of access tracts, on the presence of hydronephrosis or infection, and on surgeon experience, as well as on the efficiency with which the stone is fragmented and evacuated. Among the technical factors that the surgeon can modify at the time of surgery, the efficiency of stone fragmentation depends heavily on the intracorporeal lithotripter used. Several energy sources, including pneumatic, ultrasonic, laser, electrohydraulic and combined devices, are available, and they differ appreciably in terms of the stone clearance rate, fragmentation speed, irrigation demand and blood loss [8,9]. Selecting an energy source that maximizes clearance while limiting operative time and bleeding is therefore a central technical decision in PCNL. Pneumatic lithotripters fragment calculi through the ballistic impact of a rigid probe driven by compressed air; they fracture even hard stones with little thermal injury, but although small fragments can be washed out along the Amplatz sheath, larger fragments generally have to be retrieved individually with grasping forceps or a retrieval basket, which is time-consuming, and the ballistic energy can retropulse stones into inaccessible calyces [9,10]. In contrast, ultrasonic devices transmit vibratory energy along a hollow probe and permit simultaneous suction of debris; however, fragmentation can be slow for large or dense stones, and continuous irrigation is required to prevent the probe from overheating [11,12].

To combine the fragmentation power of ballistic energy with the aspiration capability of ultrasound, single-probe dual-energy lithotripters were developed. The ultrasonic-with-ballistic system introduced in 2017 delivers continuous ultrasonic energy together with intermittent ballistic shockwaves through one probe while maintaining suction, allowing rapid fragmentation and simultaneous clearance [13,14]. Bench and early clinical studies suggest that such combined devices fragment and evacuate stones faster than either modality alone [15-20], and comparative studies of ultrasonic, pneumatic, and combined lithotripters have generally reported comparable safety with faster clearance for combined systems [21,22]. Direct head-to-head comparisons between ultrasonic-with-ballistic devices and standard pneumatic lithotripters nevertheless remain scarce. A single randomized study reported shorter fragmentation and operative times with the combined device despite similar stone-free rates [23], and trials of newer single-probe platforms and pooled analyses have reached broadly similar conclusions [24-26]. Evidence generated in South Asia, and particularly in Bangladeshi settings, is limited and is largely extrapolated from other populations.

PCNL practices in Bangladesh must contend with high stone burdens and resource constraints, and no head-to-head comparison of these two lithotripters has been undertaken at our institution. Accordingly, the present study compared the effectiveness and safety of ultrasonic-with-ballistic lithotripsy and pneumatic lithotripsy during PCNL for renal calculi of 2-4 cm in a tertiary-care hospital. The prespecified primary outcome was the stone-free rate at four weeks; stone disintegration time, total operative time, perioperative hemoglobin drop, postoperative complications, and length of hospital stay were secondary outcomes. The aim was to provide context-specific evidence on clinical performance to inform the selection of intracorporeal lithotripters in comparable low-resource urological settings.

## Materials and methods

Study design and setting

This prospective, non-randomized quasi-experimental comparative study was carried out in the Department of Urology, Chittagong Medical College Hospital, Chattogram, Bangladesh, between January 2022 and December 2022. Treatment was allocated by alternation rather than by randomization, and the study is therefore reported as a non-randomized comparative evaluation in accordance with the TREND statement. The participant flow is shown in Figure 1.

**Figure 1:**
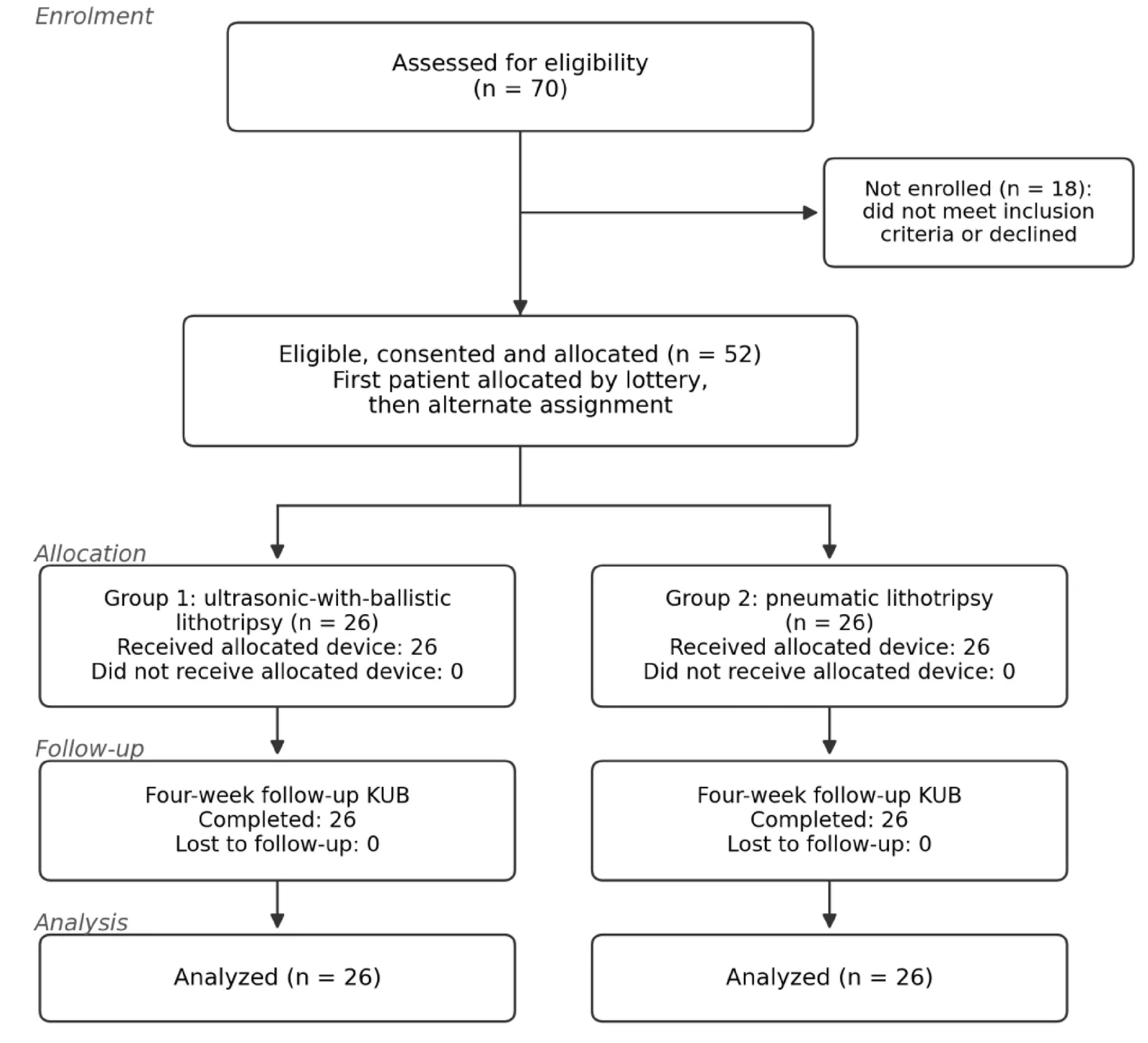
Participant flow

Participants and grouping

Adults aged 18 to 70 years with renal calculi of 2 to 4 cm who were scheduled for PCNL were enrolled by purposive consecutive sampling. Stone size was defined as the maximum diameter of the largest single calculus on preoperative imaging. Patients who also had smaller calculi in the same kidney were eligible, and five patients in each group had multiple calculi. In these patients, all stones in the kidney were treated in the same session through the same access, and stone-free status was assessed for the whole kidney. Patients were excluded if they had an isolated or obstructing ureteric stone below the upper third of the ureter, a ureteric stricture, a solitary kidney, a renal malformation, an anatomical abnormality preventing prone positioning, previous open renal surgery, impaired renal function (serum creatinine ≥1.5 mg/dL) or obesity.

The stone location was recorded as the upper ureter in three patients in Group 1 and one patient in Group 2. These patients did not have isolated ureteric stones. In each of them, the main stone was in the kidney, and part of the same stone burden extended into the pelviureteric junction or the uppermost ureter. This part was removed through the same PCNL tract during the same operation, and no patient needed a separate ureteric procedure. No patient with an isolated ureteric stone was enrolled.

Of the 70 patients approached, 52 met the eligibility criteria and gave written consent; 18 were not enrolled because they did not meet the inclusion criteria or declined participation. Participants were enrolled by the attending urology registrar on the day before surgery. The first eligible patient was allocated by lottery, and each subsequent patient was assigned alternately to the two arms by the same registrar from the enrolment register, allocating 26 patients to ultrasonic-with-ballistic lithotripsy (Group 1) and 26 to pneumatic lithotripsy (Group 2). Because the sequence alternated, the next assignment was predictable once the previous allocation was known. Allocation was therefore quasi-random rather than random; an allocation-concealment mechanism was not used, and neither the patients nor the operating surgeons were blinded to the device. Treatment allocation was determined solely by position in the enrolment sequence and never by stone characteristics, patient preference, or device availability, and the allocated lithotripter was used in every case. No eligible patient was excluded, withdrawn, or reassigned after allocation; all 52 allocated patients completed the operation and the 4-week radiographic follow-up and were analyzed in the group to which they had been allocated.

The sample size was calculated with the two-proportion formula:

\( n = \frac{2p(1-p)\left(Z_{1-\alpha/2} + Z_{1-\beta}\right)^2}{\delta_0^2} \)

where n denotes the required number of participants per group; α is the two-sided Type I error probability (0.05); β is the Type II error probability (0.05); Z(1−α/2)=1.96 is the standard normal deviate corresponding to 95% confidence; Z(1−β)=1.64 is the standard normal deviate corresponding to 95% power; p is the anticipated failure proportion (0.147) reported for pneumatic lithotripsy by Rajeev et al. [27]; (1−p) is the complementary proportion; and δ₀ is the prespecified standardized effect size (0.40). The calculation yielded 20.3 patients per group; adjustment for potential 20% attrition (20.3/0.80=25.4) and upward rounding produced a target of 26 patients per group. Accordingly, 26 patients were included in each group (total N=52).

Characterization of stone complexity

Stone burden was characterized prospectively by the maximum stone diameter measured on preoperative imaging, the number of stones, the laterality, and the stone location. Stone volume and surface area, stone attenuation in Hounsfield units, complete or partial staghorn configuration, the number of involved calyces, the degree of hydronephrosis, stone composition, preoperative urine culture and infection status, previous stone procedures and validated complexity scores such as the Guy’s Stone Score and the S.T.O.N.E. nephrolithometry score were not recorded in the study proforma and are therefore not available for analysis. Each of these variables can influence fragmentation time, operative duration, access requirements and stone-free status, and their absence is acknowledged in the Limitations section as an important source of residual confounding.

Surgical procedure

All procedures were performed under general anesthesia after antibiotic prophylaxis. A ureteric catheter was placed, the patient turned prone, and the pelvicalyceal system was opened. Fluoroscopy-guided triangulation puncture was followed by serial tract dilatation and placement of a 24-30 Fr Amplatz sheath, and nephroscopy was performed with a 24 Fr nephroscope. In Group 1, stones were fragmented with a 3.4 mm single-probe dual-energy ultrasonic-with-ballistic probe and fragments were aspirated continuously through the hollow probe; in Group 2, a 1.6 mm pneumatic probe was used and fragments were retrieved with grasping forceps. A ureteric stent was placed at the end of the procedure in both groups.

The same nephroscope, the same range of sheath sizes, and the same access technique were used in both arms, so the intracorporeal lithotripter was the only planned difference between the groups. Irrigation was with normal saline in both arms. The make, model and energy settings of the two lithotripters were not recorded in the study proforma and are therefore not reported here. This limits exact technical replication of the procedures, and it should be borne in mind when our operative and fragmentation times are compared with those of series using named devices at stated settings.

The operations were performed by more than one consultant urologist of the same unit, all of whom were already using both lithotripters in routine practice before the study period began. The number of operating surgeons, their individual case volumes, and the distribution of cases between the two arms were not recorded in the study proforma and cannot be reported. Operative time, fragment retrieval and stone-free status are strongly operator-dependent, so surgeon-related confounding and possible learning-curve effects cannot be excluded, and both are stated in the Limitations.

Outcome measures

The prespecified primary outcome was the stone-free rate at four weeks. A patient was classified as stone-free when plain radiography of the kidneys, ureters and bladder showed either no residual fragment or a single non-obstructing residual fragment smaller than 4 mm that was not associated with infection, that is, a clinically insignificant residual fragment. The term stone-free rate, according to this definition, is used throughout in place of the phrase complete stone clearance used in the original report, because a patient with a residual 3 mm fragment is not literally free of all stone material. Stone-free status was recorded against this 4 mm threshold, so patients with no visible residual fragment cannot be separated from patients with a residual fragment of 4 mm or smaller, and the two are necessarily reported together. The manuscript should not be read as reporting radiographic absence of all stone material in the patients classified as stone-free.

Two features of this assessment limit it and are stated here rather than left to be inferred. The radiopacity of each stone on preoperative imaging was not recorded in the study proforma, so it cannot be confirmed that plain radiography was equally sensitive in every patient, and a faintly opaque or radiolucent residual fragment would not have been detected. Blinding of the assessor was not part of the study design, so knowledge of the allocated device cannot be excluded as an influence on the radiographic assessment. No patient underwent computed tomography at follow-up. The consequences of assessing stone-free status radiographically rather than tomographically are discussed below and are named in the Limitations as the most important constraint on the principal finding of this study.

Secondary outcomes were the stone disintegration time, total operative time, perioperative hemoglobin drop, blood transfusion requirement, postoperative complications, and length of hospital stay. The total operative time comprised access, lithotripsy, fragment retrieval, stenting and tube placement, and was measured from the first percutaneous puncture to the end of the procedure. The stone disintegration time comprised both fragmentation and fragment extraction. Because the pneumatic arm required repeated withdrawal of the nephroscope for grasper extraction whereas the combined probe aspirated fragments as it fragmented them, this interval is more accurately described as a fragmentation-and-extraction time and reflects the efficiency of the whole stone-clearance workflow rather than fragmentation power alone; it is interpreted on that basis throughout. The decrease in hemoglobin, the difference between the preoperative value and the value on the first postoperative day, served as an indicator of blood loss.

Perioperative blood transfusion was guided by the intraoperative estimated blood loss together with hemodynamic status and any preexisting anemia, rather than by a fixed postoperative hemoglobin threshold, in keeping with departmental practice at the time. A written transfusion protocol with numerical trigger values was not applied, and the preoperative and post-transfusion hemoglobin values, the number of units given to each transfused patient, and the timing of transfusion relative to the first postoperative blood sample were not recorded in the study proforma and cannot be reported. Because transfusion may therefore have been given before the first postoperative sample was drawn, the recorded hemoglobin drop is likely to understate the true perioperative blood loss in transfused patients, and this is taken into account in the Discussion. Postoperative complications were graded via the modified Clavien-Dindo classification [28].

Statistical analysis

The data were analyzed via IBM SPSS version 23 (IBM Corp., Armonk, NY, USA). Categorical variables were summarized as frequencies and percentages, and continuous variables were summarized as means with standard deviations. Categorical variables were compared via the Pearson chi-square test, or via Fisher’s exact test for 2 × 2 tables and the Fisher-Freeman-Halton exact test for larger tables when more than 20% of cells had an expected count below 5, and continuous variables were compared via Student’s t-test for independent samples. Because a p value alone conveys little about either the size or the precision of a difference in a sample of this size, effect estimates are reported alongside the p values. Differences between proportions are presented as absolute risk differences with 95% confidence intervals calculated by the Newcombe hybrid-score method, and for the primary outcome, as a relative risk with a 95% confidence interval, while single proportions are presented with Wilson score intervals. Differences between means are presented as mean differences with 95% confidence intervals and as Cohen’s d. A multivariable model adjusting for stone size and other markers of stone complexity was considered and deliberately not fitted, because with 52 patients, 44 stone-free outcomes and only 8 patients not achieving the primary outcome, such a model would be unstable and prone to overfitting, and would give a false impression of precision. The potential for residual confounding is therefore addressed by explicit acknowledgement in the Limitations rather than by statistical adjustment. All tests were two-sided, and p <0.05 was considered statistically significant. Because the study was powered for the primary outcome only, the secondary comparisons were not adjusted for multiplicity and are regarded as exploratory.

Ethical considerations

The study was approved by the Ethical Review Committee of Chittagong Medical College (Memo No. CMC/PG/2021/800). Written informed consent was obtained from every participant; participation was voluntary, with the right to withdraw at any time, and confidentiality was maintained throughout.

## Results

The two arms were similar at baseline. Mean age was almost identical (45.4 ± 12.8 versus 45.6 ± 8.9 years; p = 0.959), and both groups had a male preponderance (61.5% and 65.4%). Right-sided stones predominated and single stones accounted for approximately four-fifths of cases in each group. Mean stone size was 2.4 mm smaller in Group 1 (26.4 ± 4.1 versus 28.8 ± 4.9 mm; mean difference −2.4 mm, 95% CI −4.9 to 0.1; p = 0.068). This difference did not reach statistical significance, but it does not establish that the two groups carried an equivalent stone burden, and an imbalance in this direction could contribute to shorter operative times in Group 1. The distribution of stone location, most commonly the renal pelvis, did not differ significantly between the groups (Fisher-Freeman-Halton exact test, p = 0.424) (Table 1).

**Table 1:** Baseline demographic and stone characteristics of the two groups Values are n (%) unless stated otherwise. The test applied to each variable is shown in the table. t, independent-samples Student’s t-test statistic; χ², Pearson chi-square statistic; NA, not applicable, because the exact test does not yield a conventional test statistic. The distribution of stone locations was compared with the Fisher–Freeman–Halton exact test rather than the Pearson chi-square test because more than 20% of cells had an expected count below five; the Pearson statistic for the same table is χ² = 4.20 with 4 degrees of freedom (p = 0.380). †These four patients had a calculus at the pelvi-ureteric junction or the proximal-most ureter contiguous with the renal stone burden, cleared antegradely through the same tract at the same sitting; no patient with an isolated ureteric stone was enrolled. The mean stone size was 2.4 mm smaller in Group 1 (mean difference −2.4 mm, 95% CI −4.9 to 0.1; Cohen’s d = −0.53); the absence of a statistically significant difference does not establish equivalence.

Characteristic	Group 1 US+ballistic (n=26)	Group 2 Pneumatic (n=26)	Test	Test statistic	p value
Age (years), mean ± SD	45.4 ± 12.8	45.6 ± 8.9	Independent t test	t = −0.052	0.959
Sex					
Male	16 (61.5)	17 (65.4)	Pearson χ²	χ² = 0.083	0.773
Female	10 (38.5)	9 (34.6)			
Stone laterality					
Right	18 (69.2)	15 (57.7)	Pearson χ²	χ² = 0.745	0.388
Left	8 (30.8)	11 (42.3)			
Stone number					
Single	21 (80.8)	21 (80.8)	Pearson χ²	χ² = 0.000	1
Multiple	5 (19.2)	5 (19.2)			
Stone size (mm), mean ± SD	26.4 ± 4.1	28.8 ± 4.9	Independent t test	t = −1.865	0.068
Stone location					
Upper calyx	2 (7.7)	0 (0)	Fisher–Freeman–Halton exact	NA	0.424
Lower calyx	5 (19.2)	4 (15.4)			
Pelvis	9 (34.6)	14 (53.8)			
Pelvicalyceal	7 (26.9)	7 (26.9)			
Upper ureter†	3 (11.5)	1 (3.8)			

Percutaneous access was comparable between the groups. A single tract sufficed in 25 patients (96.2%) in Group 1 and 24 (92.3%) in Group 2, and multiple tracts were required in one and two patients, respectively (p = 1.0). The lower calyx was the preferred puncture site in both arms, used in 19 patients (73.1%) in Group 1 and 23 (88.5%) in Group 2, with the remaining punctures distributed across the upper calyx, the middle calyx, and multiple calyces. The distribution of puncture sites did not differ significantly between the groups (χ² = 4.383; p = 0.223). Access therefore did not differ in any way that would account for the operative differences described below (Table 2).

**Table 2:** Percutaneous access characteristics of the two groups Values are n (%). The test applied to each variable is shown in the table. χ², Pearson chi-square statistic; NA, not applicable because Fisher's exact test does not provide a conventional test statistic.

Characteristic	Group 1 US+ballistic (n=26)	Group 2 Pneumatic (n=26)	Test	Test statistic	p value
Number of tracts					
Single	25 (96.2)	24 (92.3)	Fisher exact	NA	1
Multiple	1 (3.8)	2 (7.7)			
Puncture site					
Upper calyx	1 (3.8)	0 (0)	Pearson χ²	χ² = 4.383	0.223
Middle calyx	5 (19.2)	1 (3.8)			
Lower calyx	19 (73.1)	23 (88.5)			
Multicalyceal	1 (3.8)	2 (7.7)			

The two techniques differed markedly in operative efficiency and stone clearance. Total operative time was almost 30 minutes shorter with the combined device, averaging 57.7 ± 15.6 minutes in Group 1 against 86.2 ± 13.9 minutes in Group 2 (mean difference −28.5 minutes, 95% CI −36.7 to −20.3; p < 0.001). Stone disintegration time, which comprised both fragmentation and fragment extraction, was likewise shorter (22.1 ± 5.9 versus 28.9 ± 8.6 minutes; mean difference −6.8 minutes, 95% CI −10.9 to −2.7; p = 0.002). The stone-free rate at four weeks was 25 of 26 (96.2%) in Group 1 and 19 of 26 (73.1%) in Group 2 (Fisher exact p = 0.049), so a residual fragment larger than 4 mm persisted in one patient in Group 1 and in seven patients in Group 2. An additional procedure was required in one patient in each group (Table 3).

**Table 3:** Operative effectiveness outcomes of the two groups The test applied to each variable is shown in the table. t, independent-samples Student’s t-test statistic; NA, not applicable because Fisher’s exact test does not provide a conventional test statistic; —, no separate test applied, because the row is a breakdown of the primary outcome shown above it. Stone-free status was recorded against a 4 mm threshold, so patients with no visible fragment are not distinguishable from patients with a fragment of 4 mm or smaller and are counted together in the stone-free row.

Characteristic	Group 1 US+ballistic (n=26)	Group 2 Pneumatic (n=26)	Test	Test statistic	p value
Total operative time (min), mean ± SD	57.7 ± 15.6	86.2 ± 13.9	Independent t test	t = −6.955	<0.001
Stone disintegration time (min), mean ± SD	22.1 ± 5.9	28.9 ± 8.6	Independent t test	t = −3.325	0.002
Stone-free at 4 weeks (no fragment, or a single non-obstructing fragment <4 mm), n (%)	25 (96.2)	19 (73.1)	Fisher exact	NA	0.049
Residual fragment >4 mm at 4 weeks (not stone-free), n (%)	1 (3.8)	7 (26.9)	—	—	—
Additional procedure needed, n (%)	1 (3.8)	1 (3.8)	Fisher exact	NA	1

No statistically significant difference was detected in any safety outcome. Postoperative fever, the most common complication, occurred in 4 patients (15.4%) in Group 1 and 3 (11.5%) in Group 2 (p = 0.668) and resolved with antibiotics and supportive care. Blood transfusion was required in 9 patients (34.6%) and 12 patients (46.2%), respectively (p = 0.397), and the mean hemoglobin drop was modest and similar in both arms (1.4 ± 0.4 versus 1.6 ± 0.5 g/dL; t = −1.593; p = 0.118). One patient in Group 1 developed a urinary leak that resolved after repositioning of the ureteric stent and was graded as modified Clavien-Dindo IIIa. No complication of grade IIIb or above occurred, and there was no procedure-related death. Most patients in both groups were discharged on the second postoperative day (92.3% versus 88.5%; p = 1.0) (Table 4).

**Table 4:** Safety outcomes of the two groups, with complications graded by the modified Clavien–Dindo system Values are n (%) unless stated otherwise. t, independent-samples Student's t-test statistic; χ², Pearson chi-square statistic; NA, not applicable because Fisher's exact test does not provide a conventional test statistic

Characteristic	Group 1 US+ballistic (n=26)	Group 2 Pneumatic (n=26)	Test	Test statistic	p value
Fever (grade I), n (%)	4 (15.4)	3 (11.5)	Pearson χ²	χ² = 0.184	0.668
Blood transfusion (grade II), n (%)	9 (34.6)	12 (46.2)	Pearson χ²	χ² = 0.717	0.397
Urinary leakage/urinoma (grade IIIa), n (%)	1 (3.8)	0 (0)	Fisher exact	NA	1
Hemoglobin drop (g/dL), mean ± SD	1.4 ± 0.4	1.6 ± 0.5	Independent t test	t = −1.593	0.118
Hospital stays					
Hospital stay = 2 days, n (%)	24 (92.3)	23 (88.5)	Fisher exact	NA	1
Hospital stay > 2 days, n (%)	2 (7.7)	3 (11.5)	Fisher exact	NA	1

Expressing these comparisons as effect estimates rather than as p values alone shows how much uncertainty remains at this sample size (Table 5). The two time outcomes were estimated with reasonable precision, and both intervals lay entirely away from zero, the mean difference being −28.5 minutes (95% CI −36.7 to −20.3) for total operative time and −6.8 minutes (95% CI −10.9 to −2.7) for stone disintegration time, with Cohen's d values of −1.93 and −0.92. The stone-free advantage was 23.1 percentage points (95% CI 3.0 to 42.5) with a relative risk of 1.32 (95% CI 1.03 to 1.68), so the interval is wide, and its lower limit approaches the null. Every safety interval spanned 0 and remained compatible with clinically important differences in either direction, the risk difference being −11.5 percentage points (95% CI −35.4 to 14.3) for transfusion, 3.8 percentage points (95% CI −15.9 to 23.5) for fever, and −3.8 percentage points (95% CI −22.6 to 14.4) for a hospital stay beyond two days.

**Table 5:** Effect estimates with 95% confidence intervals for the principal outcomes. CI, confidence interval. Values are n (%) for categorical outcomes and mean ± SD for continuous outcomes. For categorical outcomes, the difference is the absolute risk difference in percentage points, with Newcombe hybrid-score 95% confidence intervals; for continuous outcomes, it is the mean difference in the unit shown, with 95% confidence intervals from the independent-samples t test. A positive difference favors Group 1 for the stone-free rate, and a negative difference favors Group 1 for the time, hemoglobin, transfusion, fever, and hospital-stay outcomes. The relative risk for the stone-free rate was 1.32 (95% CI 1.03 to 1.68). Cohen’s d was −1.93 for total operative time and −0.92 for stone disintegration time.

Outcome	Group 1 US+ballistic (n=26)	Group 2 Pneumatic (n=26)	Difference (95% CI)	p value
Stone-free rate at 4 weeks	25 (96.2)	19 (73.1)	23.1 (3.0 to 42.5)	0.049
Total operative time (min)	57.7 ± 15.6	86.2 ± 13.9	−28.5 (−36.7 to −20.3)	<0.001
Stone disintegration time (min)	22.1 ± 5.9	28.9 ± 8.6	−6.8 (−10.9 to −2.7)	0.002
Hemoglobin drop (g/dL)	1.4 ± 0.4	1.6 ± 0.5	−0.20 (−0.45 to 0.05)	0.118
Blood transfusion	9 (34.6)	12 (46.2)	−11.5 (−35.4 to 14.3)	0.397
Postoperative fever	4 (15.4)	3 (11.5)	3.8 (−15.9 to 23.5)	0.668
Hospital stay >2 days	2 (7.7)	3 (11.5)	−3.8 (−22.6 to 14.4)	1

## Discussion

In this prospective, non-randomized comparison of 52 patients undergoing PCNL for renal calculi of 2-4 cm, ultrasonic-with-ballistic lithotripsy was associated with shorter fragmentation-and-extraction and total operative times and with a higher radiographically defined stone-free rate than pneumatic lithotripsy, while no statistically significant difference in the observed complications was detected. The two arms were similar in the baseline characteristics that were measured, but similarity in measured characteristics does not remove confounding when allocation is not randomized, and several established determinants of PCNL outcome were not measured [8,20]. These differences are therefore reported as associations rather than as effects of the device. To our knowledge, this is among the first head-to-head comparisons of these two lithotripters from a Bangladeshi tertiary-care center.

The combined device shortened stone disintegration time (22.1 ± 5.9 versus 28.9 ± 8.6 minutes; p = 0.002) and total operative time (57.7 ± 15.6 versus 86.2 ± 13.9 minutes; p < 0.001), a reduction of practical value in high-volume units. Comparable gains have been reported in bench models [13,16], in comparative clinical series [14,18,22], in the only previous head-to-head trial against a pneumatic lithotripter [23], and in pooled analyses [25,26]. The mechanism is not fragmentation power alone. The interval recorded here included fragment evacuation, and the arms differed in how fragments were removed since the pneumatic arm required repeated nephroscope withdrawal for grasper extraction and is prone to retropulsion, whereas the combined probe aspirates continuously [10,20]. Much of the advantage therefore reflects the integrated evacuation workflow and is better described as stone-clearance efficiency than as fragmentation efficiency. The 2.4 mm smaller mean stone size in Group 1 may also have contributed.

The stone-free rate was 96.2% versus 73.1% (risk difference 23.1 percentage points, 95% CI 3.0 to 42.5; relative risk 1.32, 95% CI 1.03 to 1.68; p = 0.049). Two qualifications matter. The result is statistically fragile, because the confidence interval approaches the null and a shift of one patient between categories would cross the conventional threshold. It is also imaging-dependent. Plain radiography misses small and radiolucent fragments and overestimates stone-free rates relative to non-contrast computed tomography [21,25], and the outcome as defined permitted a non-obstructing fragment below 4 mm, so it is a stone-free rate by definition rather than radiographic absence of stone. Better clearance with combined devices has been reported repeatedly [18,22,26], whereas the one trial that used computed tomography for follow-up found no significant difference [23]. Computed tomography might therefore have narrowed the difference seen here, and this uncertainty attaches to both figures, and not only to their difference.

No excess morbidity was detected. The hemoglobin drop, transfusion requirement, and individual complication rates did not differ significantly; no complication of modified Clavien-Dindo grade IIIb or above occurred [28]; and most patients were discharged on the second postoperative day, consistent with previous comparisons across lithotripter types [22,29]. These findings must be read against the sample size, since post hoc power for the observed transfusion difference was approximately 14%, so a non-significant p value is uninformative about equivalence and the intervals in Table 5 remain compatible with clinically important differences in either direction. The transfusion rates of 34.6% and 46.2% are higher than usually reported after PCNL despite mean hemoglobin decreases of only 1.4 and 1.6 g/dL. Three factors most plausibly account for this, namely, a cautious, locally determined threshold adopted where postoperative monitoring and urgent cross-matching are limited, a high background prevalence of preoperative anemia, and transfusion given before the first postoperative sample, which makes the recorded drop an underestimate of true blood loss. The supporting hematological values were not recorded, so these remain explanations rather than findings.

This study has important limitations. Allocation was quasi-random rather than random, since after the first patient was allocated by lottery the sequence alternated predictably, no concealment mechanism was used, and neither patients nor surgeons were blinded, so selection bias cannot be excluded. Several determinants of PCNL difficulty were not captured by the study proforma, including stone volume, attenuation, staghorn configuration, hydronephrosis, and validated complexity scores, so residual confounding is possible, and the 2.4 mm difference in mean stone size shows that the arms were not demonstrably equivalent in stone burden. No adjusted analysis was fitted because with only eight failures of the primary outcome, a multivariable model would be unstable. Device settings, surgeon number and experience, stone radiopacity and the hematological detail underlying transfusion were also unrecorded, which limits technical replication and leaves surgeon-related confounding unquantified. Stone-free status was assessed radiographically rather than tomographically and against a 4 mm threshold, the single most important constraint on the principal finding. The study was powered only for a large difference in the primary outcome, not for any safety comparison, and no cost data were collected. These findings are therefore hypothesis-generating, and adequately powered multicenter randomized trials with computed tomography-based follow-up and cost-effectiveness analyses are needed.

## Conclusions

Both ultrasonic-with-ballistic and pneumatic lithotripsy are effective and safe for the management of renal calculi during PCNL. In this prospective non-randomized comparative study, ultrasonic-with-ballistic lithotripsy was associated with shorter fragmentation-and-extraction and total operative times and with a higher stone-free rate on radiographic assessment than pneumatic lithotripsy, while the observed complications and the length of hospital stay did not differ significantly, although the study was not powered to establish equivalence in safety. Given the small sample, the non-randomized allocation, the limitations of radiographic follow-up, and the absence of any economic evaluation, these findings describe an association rather than a causal effect and do not, by themselves, justify a recommendation for routine adoption of the combined device. They should be confirmed in adequately powered multicenter randomized trials using standardized computed tomography-based stone-free assessment, and cost-effectiveness should be evaluated before the combined lithotripter is recommended for routine use in resource-limited settings.

## References

[1] Akram M, Somani B (2025). Epidemiology and management of kidney stone disease - current insights. Res Rep Urol.

[2] Fernström I, Johansson B (1976). Percutaneous pyelolithotomy. A new extraction technique. Scand J Urol Nephrol.

[3] Ganpule AP, Vijayakumar M, Malpani A, Desai MR (2016). Percutaneous nephrolithotomy (PCNL) a critical review. Int J Surg.

[4] Sabler IM, Katafigiotis I, Gofrit ON, Duvdevani M (2018). Present indications and techniques of percutaneous nephrolithotomy: what the future holds?. Asian J Urol.

[5] Knoll T, Daels F, Desai J (2017). Percutaneous nephrolithotomy: technique. World J Urol.

[6] Wei C, Zhang Y, Pokhrel G (2018). Research progress of percutaneous nephrolithotomy. Int Urol Nephrol.

[7] Ibrahim A, Wollin D, Preminger G, Andonian S (2018). Technique of percutaneous nephrolithotomy. J Endourol.

[8] Scotland KB, Kroczak T, Pace KT, Chew BH (2017). Stone technology: intracorporeal lithotripters. World J Urol.

[9] Wollin DA, Lipkin ME (2019). Emerging technologies in ultrasonic and pneumatic lithotripsy. Urol Clin North Am.

[10] Radfar MH, Basiri A, Nouralizadeh A (2017). Comparing the efficacy and safety of ultrasonic versus pneumatic lithotripsy in percutaneous nephrolithotomy: a randomized clinical trial. Eur Urol Focus.

[11] Khoder W, Strittmatter F, Alghamdi A, Seitz M, Stief C, Bader MJ (2020). Comparative evaluation of tissue damage induced by ultrasound and impact dual-mode endoscopic lithotripsy versus conventional single-mode ultrasound lithotripsy. World J Urol.

[12] Borofsky MS, El Tayeb MM, Paonessa JE, Lingeman JE (2015). Initial experience and comparative efficacy of the UreTron: a new intracorporeal ultrasonic lithotriptor. Urology.

[13] Carlos EC, Wollin DA, Winship BB (2018). In vitro comparison of a novel single probe dual-energy lithotripter to current devices. J Endourol.

[14] Chew BH, Matteliano AA, de Los Reyes T, Lipkin ME, Paterson RF, Lange D (2017). Benchtop and initial clinical evaluation of the ShockPulse stone eliminator in percutaneous nephrolithotomy. J Endourol.

[15] Axelsson TA, Cracco C, Desai M (2021). Consultation on kidney stones, Copenhagen 2019: lithotripsy in percutaneous nephrolithotomy. World J Urol.

[16] Auge BK, Lallas CD, Pietrow PK, Zhong P, Preminger GM (2002). In vitro comparison of standard ultrasound and pneumatic lithotrites with a new combination intracorporeal lithotripsy device. Urology.

[17] Hofmann R, Weber J, Heidenreich A, Varga Z, Olbert P (2002). Experimental studies and first clinical experience with a new lithoclast and ultrasound combination for lithotripsy. Eur Urol.

[18] Pietrow PK, Auge BK, Zhong P, Preminger GM (2003). Clinical efficacy of a combination pneumatic and ultrasonic lithotrite. J Urol.

[19] Cho CO, Yu JH, Sung LH, Chung JY, Noh CH (2010). Comparison of percutaneous nephrolithotomy using pneumatic lithotripsy (Lithoclast®) alone or in combination with ultrasonic lithotripsy. Korean J Urol.

[20] Lowe G, Knudsen BE (2009). Ultrasonic, pneumatic and combination intracorporeal lithotripsy for percutaneous nephrolithotomy. J Endourol.

[21] Karakan T, Diri A, Hascicek AM, Ozgur BC, Ozcan S, Eroglu M (2013). Comparison of ultrasonic and pneumatic intracorporeal lithotripsy techniques during percutaneous nephrolithotomy. ScientificWorldJournal.

[22] Zengin K, Sener NC, Bas O, Nalbant I, Alisir I (2014). Comparison of pneumatic, ultrasonic and combination lithotripters in percutaneous nephrolithotripsy. Int Braz J Urol.

[23] Yadav BK, Basnet RB, Shrestha A, Shrestha PM (2021). Comparison between shockpulse and pneumatic lithotripsy in percutaneous nephrolithotomy. World J Urol.

[24] Large T, Nottingham C, Brinkman E (2021). Multi-institutional prospective randomized control trial of novel intracorporeal lithotripters: Shockpulse-SE vs Trilogy trial. J Endourol.

[25] Mykoniatis I, Pyrgidis N, Tzelves L (2023). Assessment of single-probe dual-energy lithotripters in percutaneous nephrolithotomy: a systematic review and meta-analysis of preclinical and clinical studies. World J Urol.

[26] Lin L, Zhou L, Xiao K (2022). Does combined lithotripter show superior stone-success rate than ultrasonic or pneumatic device alone during percutaneous nephrolithotrotomy? A meta-analysis. Int J Surg.

[27] Rajeev T, Pratihar SK, Sarma D, Baruah SJ, Barua SK, Bagchi PK, Phukan M (2020). A comparative study between holmium laser, pneumatic lithotripsy and shock pulse in terms of efficacy and safety in percutaneous nephrolithotomy (PCNL): a prospective randomized study. J Endolum Endourol.

[28] Dindo D, Demartines N, Clavien PA (2004). Classification of surgical complications: a new proposal with evaluation in a cohort of 6336 patients and results of a survey. Ann Surg.

[29] Krambeck AE, Miller NL, Humphreys MR (2011). Randomized controlled, multicentre clinical trial comparing a dual-probe ultrasonic lithotrite with a single-probe lithotrite for percutaneous nephrolithotomy. BJU Int.

